# BitMapper: an efficient all-mapper based on bit-vector computing

**DOI:** 10.1186/s12859-015-0626-9

**Published:** 2015-06-11

**Authors:** Haoyu Cheng, Huaipan Jiang, Jiaoyun Yang, Yun Xu, Yi Shang

**Affiliations:** 1 Key Laboratory on High Performance Computing, Hefei, Anhui230027, P.R. China; 2 School of Computer Science, University of Science and Technology of China, Hefei, Anhui, 230027 P.R. China; 3 Hefei University of Technology, Hefei, 230009 China; 4 Department of Computer Science, University of Missouri-Columbia, Columbia MO, 65203 USA

**Keywords:** Read alignment, Edit distance, Multiple locations, Simultaneously calculating, Bit-vector algorithm

## Abstract

**Background:**

As the next-generation sequencing (NGS) technologies producing hundreds of millions of reads every day, a tremendous computational challenge is to map NGS reads to a given reference genome efficiently. However, existing methods of all-mappers, which aim at finding all mapping locations of each read, are very time consuming. The majority of existing all-mappers consist of 2 main parts, filtration and verification. This work significantly reduces verification time, which is the dominant part of the running time.

**Results:**

An efficient all-mapper, BitMapper, is developed based on a new vectorized bit-vector algorithm, which simultaneously calculates the edit distance of one read to multiple locations in a given reference genome. Experimental results on both simulated and real data sets show that BitMapper is from several times to an order of magnitude faster than the current state-of-the-art all-mappers, while achieving higher sensitivity, i.e., better quality solutions.

**Conclusions:**

We present BitMapper, which is designed to return all mapping locations of raw reads containing indels as well as mismatches. BitMapper is implemented in C under a GPL license. Binaries are freely available at http://home.ustc.edu.cn/%7Echhy.

**Electronic supplementary material:**

The online version of this article (doi:10.1186/s12859-015-0626-9) contains supplementary material, which is available to authorized users.

## Background

Recently, DNA sequencing has become a powerful tool for researches in biology and medicine. The decreasing cost and improving speed of the next-generation sequencing (NGS) technologies generate massive reads every day. However, a disadvantage of NGS technologies is that they produce sequenced reads of relatively short length. For instance, the HiSeq2500 platform of Illumina usually produces 150 bp reads. The first step of many genomic researches is finding the mapping locations of these short NGS reads in a given large reference genome.

For this mapping issue, two classes of methods have been developed. One class, including Bowtie [1], Bowtie2 [2], BWA [3], GEM [4], etc., is referred to as best-mappers for trying to identify one or a few best mapping locations for each read. The other class, including RazerS 3 [5], Hobbes2 [6], and mrFAST [7, 8], is referred to as all-mappers for finding all mapping locations. Generally, the selection of different mappers depends on the needs of down-stream applications. Finding one or a few best mapping locations for each read using best-mappers is enough in most cases (e.g., mapping DNA-protein interactions, whole-transcriptome sequencing and whole genome expression profiling). However, for some specific applications, such as ChIP-seq experiments, CNVs (copy number variation) calling and detecting structural variants, it is necessary to identify all mapping locations using all-mappers.

Due to the different purposes, identifying all mapping locations using all-mappers is usually much slower than finding one or a few best locations using best-mappers. An important reason is that all-mappers have to enumerate all possible locations, while best-mappers can use some heuristic methods to select the most likely one. There are a lot of matches for some reads due to huge numbers of segmental duplications and common repeats in reference genomes. Thus, finding all mapping locations is still a computationally very expensive problem.

To solve this problem, many all-mappers have been developed. Most of them consist of two parts, filtration and verification. Filtration reduces the number of the locations that need to be verified (called candidates), especially when a reference genome is extremely large. For example, Hobbes [9] uses a dynamic programming algorithm to select several *q*-grams with the lowest frequency, where *q*-grams are the subsequences with length of *q*. Therefore, the number of candidates is minimal. Another filter proposed by Hobbes 2 chooses *k*+2 *q*−grams instead of *k*+1 and only verifies the locations that appear at least two times. Recently, Masai [10] improved the performance of filtration by generating candidate locations of multiple reads simultaneously and using approximate seeds. Compared with filtration, verification used for edit distance is the dominant part of the whole running time in current mappers [8]. Several algorithms have been proposed to speedup verification. A bit-vector algorithm proposed by Myers [11] uses bit representation contained in a machine word to calculte edit distance. RazerS 3 [5] implements a banded version of Myers’ algorithm [12], which only calculates several consecutive diagonals rather than the whole dynamic programming matrix. Although the current banded method of verification is quite quick, it only calculates the edit distance of a read to one location rather than multiple locations.

In this paper, we present BitMapper, an efficient read mapper which is designed to return all mapping locations of raw reads containing indels as well as mismatches. It includes a new vectorized bit-vector algorithm using a single machine word to represent several bit vectors and simultaneously calculates the edit distance of a read to multiple locations in a given reference genome. A vectorized verification scheme is also proposed to work with the new bit-vector algorithm. Experimental results show that the running time of BitMapper is from several times to an order of magnitude faster than the best existing all-mappers, including Hobbes 2, RazerS 3, mrFAST (with FastHASH) [8], Masai and Yara [13].

## Methods

First, we define the read mapping problem and related concepts.

### **Definition1**.

Given a set of reads R and a reference genome S, find all locations in S where the hamming or edit distance of each read in R is at most k.

Hamming and edit distance are two common distance metrics for sequence alignment. Hamming distance only includes the substitutions of the corresponding symbols between two strings of equal length, while edit distance consists of substitutions, insertions and deletions. Calculating hamming distance is relatively easy and has been well solved. On the other hand, calculating edit distance efficiently is still difficult, which is the focus on this article.

Similar to existing short reads mappers, BitMapper mainly consists of two parts: filtration and verification. In the following, we first briefly describe the procedure of existing approaches and then present and analyze BitMapper in detail.

### Filtration

Filtration is an important phase for sequence alignment, especially if a reference genome is extremely large. Only the regions consisting of potential mapping locations can be reserved after filtration. Currently, the basic principle of nearly all *q*-gram-based filtration strategies is that the number of *q*-grams shared between two sequences should exceed a certain threshold if they are potentially similar. Next, we briefly summarize commonly used*q*-gram-based approaches.

#### Pigeonhole principle

A simple and efficient filtration strategy is pigeonhole principle: if *l* items are put into *l*+1 boxes, then one or more boxes would be empty. In its application on sequence alignment, first each read is divided into *k*+1 non-overlapping *q*-grams, where *k* is the threshold of edit distance or hamming distance. If the distance between a read and a candidate region is less than *k*, at least one in *k*+1 non-overlapping *q*-grams of the read can be mapped to the reference exactly, since a substitution, insertion or deletion only affects a *q*-gram. A more general version of pigeonhole principle is that if a read is able to be cut into *k*+*m* non-overlapping *q*-grams, sharing at least *m* of them with a read is necessary for each mappinglocation.

#### Count filtering

Compared with pigeonhole principle, a more involved filtration strategy is count filtering. Given a sequence *s*, there are |*s*|−*q*+1 overlapping *q*-grams that are obtained by sliding a window of length *q* over *s*, where |*s*| is the length of *s*. As in the explanation of pigeonhole principle, a substitution only affects at most *q* overlapping *q*-grams. Thus, no more than *k*×*q* *q*-grams could be affected with hamming distance *k*. If the hamming distance between *s* and another sequence *r* is less than *k*, then the number *T* of shared *q*-grams is at least.

(1) $$ T=|s|-(k+1)\times q+1  $$

The lower bound *T* of edit distance is similar to that of hamming distance. The first method of count filtering on sequence alignment is a modified SWIFT algorithm [14] used in RazerS [15].

#### Our implementation

Pigeonhole principle is faster than count filtering on filtration phase, while the verification time of the pigeonhole-principle-based mappers is more than that of the count-filtering-based mappers. In fact, there is a tradeoff between filtration and verification. Because the proportion of verification time for the pigeonhole-principle-based mappers is larger than that for the count-filtering-based mappers, the former benefit more from the improvement of verification than the latter. As our verification method is efficient, Bitmapper used pigeonhole principle instead of count filtering.

### Verification

The locations reserved after filtration are the candidates for matches. During the verification phase, these candidates should be verified by calculating their edit distance or hamming distance to each read. Compared with computing hamming distance, computing edit distance is extremely time-consuming. In the following, we first describe the theoretical basis for our vectorized Gene Myers’ bit-vector algorithm, and illustrate the algorithm in detail. Then, we present a vectorized verification scheme, which is designed to work with the vectorized Gene Myers’ bit-vector algorithm.

#### Theoretical basis

For sequence alignment, the reads and the reference genomes can be viewed as the strings including letters *A*, *C*, *G*, *T* and *N*. Assume the length of read *r* is *m*, the length of genome *s* is *n*, and the threshold of edit distance is *k*. The dynamic programming algorithm proposed in [16] is a classic method for this problem, which computes a dynamic programming matrix *C*[0…*m*,0…*n*] of size (*m*+1)×(*n*+1). The well-known recurrence formula is as follows.

(2) $$ {\small{\begin{aligned} &E[i,j]=\left\{ \begin{array}{ll} 0& r_{i}=s_{j}\\ 1& {r_{i}\neq s_{j}}\\ \end{array} \right.& {i \in [1,m],j \in [1,n]} & \\ &C[i,j]=min\left\{ \begin{array}{ll} C[i,j-1]+1\\ C[i-1,j]+1\\ C[i-1,j-1]+E[i,j] \end{array} \right.& {i \in [1,m],j \in [1,n]}& \\ &C[0,j]=0,C[i,0]=i & {i \in [0,m],j \in [0,n]}& \end{aligned}}}  $$

Its time complexity is *O*(*m*×*n*) and it is very slow when a reference genome is large. Actually, calculating the whole dynamic programming matrix is unnecessary when the edit distance threshold *k* has been set in advance. As stated in the following Lemma 1, the size of computing area in dynamic programming matrix is related to *k*, which has been proven in [17].

##### **Lemma1**.

Given a read of length m, a candidate location d in a reference genome and an edit distance threshold k, the start and end positions of potential matches may be from *d*−*k* to *d*+*k* and from *d*+*m*−*k*−1 to *d*+*m*+*k*−1, respectively. In other words, the length of the verification window, which would be calculated with the read, is *m*+2*k*.

**Fig. 1:**
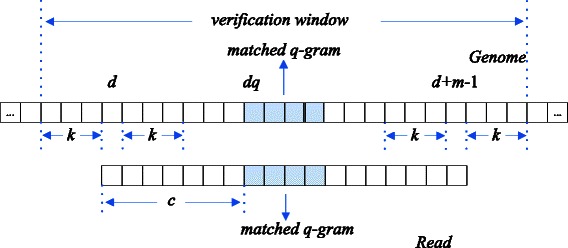
The verification window in reference genome. It includes the candidate mapping locations of the read with edit distance up to 3

Figure 1 shows an example for Lemma 1. Note that the candidate location *d* is obtained by subtracting the offset *c* from *dq*, where *dq* is an exactly matched location of a *q*-gram and *c* is the offset of this *q*-gram in the read. If there are only at most *k* deletions, the segment starting at *d*−*k* and ending at *d*+*m*+*k*−1 needs to be computed. If there are only at most *k* insertions, the segment range needing to be considered is from *d*+*k* to *d*+*m*−*k*−1. Combining with these two intervals, the range of maximal verification window is from *d*-*k* to *d*+*m*+*k*−1.

**Fig. 2:**
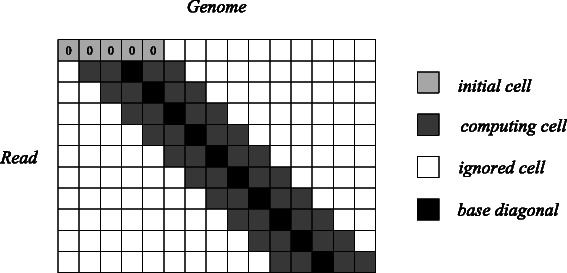
The computing area in dynamic programming matrix with the edit distance threshold k = 2. The initial cells should be set to 0 because the start locations of potential matches can not be known in advance

According to Lemma 1, the length of verification window is *m*+2*k*. Thus, only (*m*+2*k*+1)×(*m*+1) cells in dynamic programming matrix need to be calculated. We define the diagonal which is shifted from the main diagonal by *k* diagonals to the right as “base diagonal”. It corresponds to the situation that only substitutions are considered, since the computing path moves right down from the current cell in dynamic programming matrix to the adjacent cell when a substitution occurs. For a deletion in the reference genome, the computing path goes right to the adjacent cell. For an insertion, this path goes down to the adjacent cell. Thus, the rightmost and the leftmost diagonals are obtained by sliding *k* diagonals from the “base diagonal” to its right and left, respectively. In fact, the computing area in dynamic programming matrix is a banded parallelogram, as shownin Fig. 2.

An efficient solution for this problem is the bit-vector algorithm proposed in [11], which is based on the observation that the difference of the values between adjacent cells in dynamic programming matrix is at most 1. It is able to encode a whole column in dynamic programming matrix using bit vectors and compute a column by bit operations rather than cell-by-cell. Banded versions of Myers’ bit-vector algorithm have been implemented in [5] and [12]. They encode a banded parallelogram in dynamic programming matrix into columns for column-wise computation, since only limited consecutive diagonals need to be calculated rather than the whole dynamic programming matrix, according to the analysis above. Figure 2 shows that at most 2*k*+1 cells in each column need to be calculated, so that the length of the bit vectors is also 2*k*+1. If 2*k*+1 is less than the word size of computer, a column could be processed in one step.

#### Vectorized Gene Myers’ bit-vector algorithm

A significant characteristic of the NGS reads is that the length of them is relatively short. For the down-stream applications using all-mappers, the edit distance threshold is usually set to 4 % or 5 % of the read length. Thus, the edit distance threshold *k* is usually low. It means that a few bits are enough for banded bit-vector algorithms to calculate edit distance. For example, the length of the reads sequenced by Illumina platform is always under 150 so that the threshold *k* is set to 7. If *k*=7, the length of bit vectors is 15, while the word size of modern computers is typically 64 and the Streaming SIMD Extensions (SSE) instruction set has several 128-bit registers. Therefore, it is possible to load multiple bit vectors into a machine word or a 128-bit SSE register. Furthermore, the problem can be converted to how to compute the edit distance between several patterns and a text. Based on these observations, we propose a new vectorized Gene Myers’ bit-vector algorithm to simultaneously process a text with multiple patterns.

First we briefly introduce the current bit-vector algorithm proposed in [12], which is the basis of our vectorized algorithm. It uses delta encoding in dynamic programming matrix *C*[0…*m*,0…*n*]. Specifically, for column *j*, the bit delta vectors are.

(3) $$\begin{array}{*{20}l} &{HP}_{j}[i] \equiv(C[i,j]-C[i,j-1]=+1)& \\ &{HN}_{j}[i] \equiv(C[i,j]-C[i,j-1]=-1)& \\ &{VP}_{j}[i] \equiv(C[i,j]-C[i-1,j]=+1)& i \in [1,m],j \in [1,n] & \\ &{VN}_{j}[i] \equiv(C[i,j]-C[i-1,j]=-1)&\\ &D0_{j}[i]=C[i,j]-C[i-1,j-1]& \\ &{Peq}_{j}[s][i]\equiv(pattern[i]=s)& s \in \{A,T,G,C\}& \end{array} $$

where *H* *P*_*j*_,*H* *N*_*j*_,*V* *P*_*j*_,*V* *N*_*j*_,*D*0_*j*_ and *P* *e* *q*_*j*_ are the *j*th element of *H* *P*,*H* *N*,*V* *P*,*V* *N*,*D* and *P* *e* *q*, respectively. And the notation *H* *P*_*j*_[*i*],*H* *N*_*j*_[*i*],*V* *P*_*j*_[*i*],*V* *N*_*j*_[*i*],*D*0_*j*_[*i*] and *P* *e* *q*_*j*_[*s*][*i*] denote the *i*th bit of *H* *P*_*j*_,*H* *N*_*j*_,*V* *P*_*j*_,*V* *N*_*j*_,*D*0_*j*_ and *P* *e* *q*_*j*_[*s*], respectively.

If the values of these bit vectors in column *j*−1 have already been known, then the bit vectors in column *j* can be computed as following.



where *t*[*p*] is the *p*th element of text. Because the length of each column in computing area is 2*k*+1, so the length of these bit vectors is also 2*k*+1.

Based on the Algorithm 1, we developed the vectorized Gene Myers’ bit-vector algorithm. Briefly, it packs multiple bit-vectors of different patterns into a machine word so that these patterns are able to be processed with one text simultaneously. The calculating of each bit-vector is similar to the previous banded bit-vector algorithm [12]. However, some problems would occur when multiple bit-vectors are processed as a whole. From Algorithm 1, only six operations have been used: ‘ ⊕’, ‘ |’, ‘ &’, ‘ ≫’, ‘ ∼’ and ‘+’. They can be divided into two groups. The first group consists of ‘ ⊕’, ‘ |’, ‘ &’ and ‘ ∼’, while ‘ ≫’ and ‘+’ belong to the second group. Using the operations of the first group in a machine word including multiple bit vectors does not have any difficulty, because the bit vectors in it cannot be affected with each other. However, implementing the operations of the second group as a whole would influence each other. For operation ‘+’, the carries resulted from addition of lower bit vectors would affect the nearly upper bit vectors. For operation ‘ ≫’, the lowest bit in upper bit vector would move right to influence the nearly lower bit vector.

**Fig. 3:**
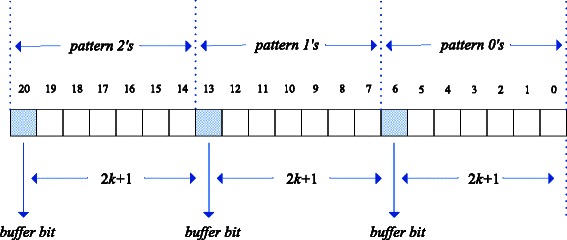
The data structure for the variables containing several bit vectors. Each pattern needs (2*k*+2) bits to represent itself

To solve the problem about operation ‘+’, the data structures of the variables used in our vectorized Gene Myers’ bit-vector algorithm have been redesigned. For the previous banded bit-vector algorithm [12], 2*k*+1 bits are enough for each variable. Intuitively, for the vectorized algorithm, the length of each variable, which represents *n* bit vectors, is (2*k*+1)×*n*. However, if multiple bit vectors are loaded into a machine word as this, the problem above could not be solved. Our solution is to use one more bit between two bit vectors as a buffer, so that the carries resulted from the operation ‘+’ among the lower bit vectors would not affect the upper bit vectors. For example, *V* *P*_*l*_, which represents the difference of values between the vertical cells for pattern *l*, is a part of *V* *P*, starting from (2*k*+2)×*l*th bit and ending at (2*k*+2)×(*l*+1)−1th bit. For *h* patterns, the bit vectors of them are assembled together so that the length of variables including *VP*, *V* *N*,*H* *P*,*H* *N*,*D*0 and *Peq* is not less than (2*k*+2)×*h*, as shown in Fig. 3. And the problem about operation ‘ ≫’ has been solved in our vectorized Gene Myers’ bit-vector algorithm by using an extra ‘ &’ operation with a predefined bitmask.

Our vectorized Gene Myers’ bit-vector algorithm proceeds column-by-column through the dynamic programming matrix. If the length of patterns is less than that of the text, it returns the optimal end location for each pattern on text. Otherwise, it returns the optimal end locations for the text on each pattern. As an example, we present the outline of the algorithm for the second situation as follows.

1. Preprocess the variables for column 0.
  Set the *Peq* array for the first 2*k*+1 symbols in each pattern.
  Set *VP*, *VN* and *E* to 0, where *E* contains the edit distances for *h* patterns.
2. Scan and compute the banded parallelogram in the dynamic programming matrix from left to right by column.
  Compute *HP*, *HN* and *D*0 of column *j* from *VP* and *VN* of column *j*-1.
  Compute *VP* and *VN* of next column using *HP*, *HN* and *D*0.
  Set *Peq* for next column by shifting to the right of the current *Peq*.
  Update *E* for this column using *D*0.
3. Output the locations with the lowest edit distance as the optimal end location on each pattern, separately. The range in *E* from (2*k*+2)×*j*th bit to (2*k*+2)×(*j*+1)−1th bit denotes the edit distance of pattern *j*.

All of the patterns have to be processed one by one in step 3, while step 1 and step 2 can process multiple patterns simultaneously. Fortunately, unlike step 1 and step 2, step 3 is not always necessary due to two reasons: a) the number of matched locations is much less than that of the candidate locations, and b) a simple branch-cut strategy is used in step 2 to stop algorithm earlier, as described in [5]. More details of the vectorized Gene Myers’ bit-vector algorithm can be found in the Additional file 1: Section S5.

#### Influence of the number of patterns

**Fig. 4:**
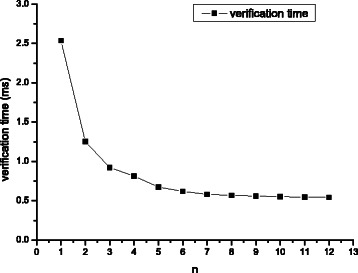
Performance for the vectorized Gene Myers’ bit-vector algorithm according to different *n*

We have already implemented the vectorized Gene Myers’ bit-vector algorithm using 64-bit machine word and 128-bit SSE2 register. It can calculate the edit distance of a text with *n* patterns. In order to figure out the influence of different *n*, we selected a 100 bp read from specimen HG00096 as a text and regarded 1 thousand subsequences of human genome starting at the candidate locations of this read as patterns. The threshold *k* of edit distance was set to 4. Because the length of each bit vector is 2*k* + 2=10 and the length of a SSE register is 128, the vectorized Gene Myers’ bit-vector algorithm can process at most 12 patterns with a text simultaneously. Figure 4 shows the running time of the algorithm with different *n*. Although the performance was improved until *n*=12, we found that the running time decreased rapidly from *n*=1 to *n*=8, while it only decreased a little from *n*=9 to *n*=12.

The reason is that for the original banded bit-vector algorithm which calculates the edit distance between a text and a pattern, algorithm stops once it meets the requirement of the branch-cut strategy. For our vectorized Gene Myers’ bit-vector algorithm, calculating stops until all of the patterns meet the requirement. It is difficult when *n* is large and would result in extra cost. Therefore, we set *n* to 8 in most cases. For higher edit distance threshold, *n* is set to 4 since a 128-bit register cannot load 8 bit vectors.

#### Vectorized verification scheme

In order to make full use of the vectorized algorithm, the patterns used to compare with a same text should be collected. The traditional verification scheme, which only selects a read and a subsequence in given reference genome as input every time, is not suitable for our vectorized Gene Myers’ bit-vector algorithm. It is necessary to propose a vectorized verification scheme that considers multiple reads as patterns and a subsequences in given reference genome as text, or vice versa. In other words, multiple reads may correspond to one location in given reference genome, or multiple locations correspond to one read.

**Fig. 5:**
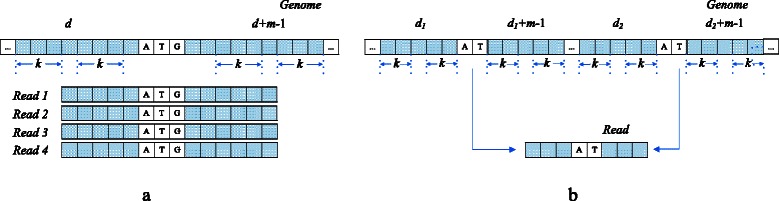
Two vectorized verification schemes for the vectorized Gene Myers’ bit-vector algorithm. **a** A location in a reference genome corresponds to four reads. **b** A read corresponds to two locations in the reference genome

Figure 5a shows the vectorized verification scheme A, which considers multiple reads as patterns and a subsequence in given reference genome as a text. All of the four reads have a matched 3-gram *ATG* in the reference genome and share the same candidate location *d*. Generally, this scheme needs to build a reads index in order to collect the reads sharing the same locations efficiently. Figure 5b shows vectorized verification scheme B that considers a read as a text and multiple subsequences starting at the candidate locations of the read as patterns. The read here corresponds to two subsequences sharing a 2-gram *AT*. These two subsequences are obtained by looking up the index of the reference genome using the non-overlapping *q*-grams of the read.

According to the analysis above, we found that scheme A takes advantage of the repeatability of the reads, while B takes advantage of the repeatability of the reference genomes. In the experimental results presented in Additional file 1: Section S2, the repeatability of genomes is much more than that of reads. Therefore, scheme B suits the vectorized Gene Myers’ bit-vector algorithm better than A. Another advantage of scheme B is that it does not need an extra index of reads. For the reasons outlined above, BitMapper is implemented as scheme B.

## Results and discussion

BitMapper was compared with five state-of-the-art all-mappers, including mrFAST (with FastHASH), Hobbes 2, RazerS 3, Masai and Yara, and three popular best-mappers, Bowtie 2, GEM and BWA in our experiments. The default configurations of these mappers were used except stated otherwise, and the results were output in the SAM format. For a fair comparison, all mappers ran on the same computer with an Intel(R) Core(TM) i7-4770 processor and 24GB of RAM running 64-bit Ubuntu 14.04.

The distance metric used in our experiments was edit distance with threshold 5 %. The reference genomes were the whole genome of human (NCBI HG19), caenorhabditis elegans (WormBase WS201) and arabidopsis thaliana (assembly TAIR10). In the following, mapping time and sensitivity on both real and simulated data sets were presented.

### Sensitivity comparison using Rabema results

In this experiment, 100 k simulated 100 bp reads of human were generated by a simulator tool Mason [18] using default profile setting. And we also selected a real data set consisting of 1 million 100 bp reads from specimen HG00096 of the 1000 Genome project [19]. To compare the sensitivity of single-end alignment in different genomes, we used the first 1 million 100 bp reads of the data sets SRX026594 and the first 1 million 101 bp reads of SRR1604937, which were obtained from the DNA Data Bank of Japan (DDBJ) repository [20] and National Center for Biotechnology Information (NCBI) repository [21], respectively.

To compare the sensitivity of different mappers fairly, Rabema benchmark [22] was used to evaluate them. It has been widely used in recent articles, such as [5, 6] and [10]. The categories of sensitivity scores provided by Rabema benchmark include all, all-best, and any-best, which are designed to denote the mapped fraction of all, all of the best, and any of the best matches. And to measure these scores, Rabema benchmark defines two metrics: *normalized found interval* and *found interval*. For *normalized found interval*, each read is given at most one point no matter how many mapping locations it has. For *found interval*, each mapping location is given one point [see Additional file 1: Section S4 for more detailed illustration]. Note that we only presented the Rabema scores (*normalized found interval*) in the following, and presented the Rabema scores (*found interval*) in Additional file 1: Section S4 due to the limited space. Because Rabema benchmark needs a baseline of mapping locations to build a gold standard, we implemented RazerS 3 in full sensitivity mode, which can report 100 % of mapping locations for each read.

#### Rabema benchmark results on simulated data

Table 1 shows the results of mapping 100k simulated reads to the reference genome of human. The Rabema all, all-best and any-best scores were presented here. Each Rabema category has a large number and 6 small numbers representing the total score and the scores for mapping locations with $\binom {0\ 1\ 2}{3\ 4\ 5}$ errors, respectively. Best-mappers including Bowtie 2, GEM and BWA were implemented in both high and default sensitivity mode. In high sensitivity mode, the Rabema all-scores (*normalized found interval*) for Bowtie 2, BWA and GEM were 99.73 %, 97.80 % and 96.02 %, respectively. It seems that these best-mappers can achieve nearly full sensitivity. However, the Rabema all-scores (*found interval*) of BWA and GEM, which can be found in in Additional file 1: Table S3, were 86.67 % and 61.46 %, respectively. For Bowtie 2, although the Rabema all-scores (*found interval*) was still more than 98 %, it was extremely slow using one thread. Thus, we implemented Bowtie 2 with 16 threads and did not present its running time here. This means that the best-mappers are not suitable for applications requiring full or nearly full sensitivity. It is mainly because the best-mappers are designed specifically for identifying the best mapping locations of each read.

**Table 1:** Rabema benchmark results (*normalized found interval*) for 100 k simulated reads

Mapper	Time	Benchmark category											
	[min:sec]	All[%]				All-best[%]				Any-best[%]			
Bowtie2 ^a^	0:18	90.18	97.68	96.60	92.25	95.87	96.46	96.14	94.48	99.26	100.00	99.49	97.51
			78.95	52.71	21.13		93.69	92.76	92.27		96.63	96.27	95.38
BWA ^b^	0:49	92.28	100.00	99.81	96.75	98.84	100.00	99.80	99.40	98.89	100.00	99.86	99.50
			79.47	44.91	16.65		93.61	78.42	70.67		93.70	78.61	71.17
GEM ^c^	0:14	92.75	98.25	97.65	95.69	98.15	98.27	98.21	97.97	99.36	99.42	99.42	99.24
			88.44	67.06	33.44		98.11	96.87	95.87		99.42	98.74	97.33
Bowtie 2	—	99.73	100.00	100.00	100.00	99.97	100.00	100.00	100.00	99.97	100.00	100.00	100.00
			99.96	99.53	95.45		99.95	99.67	97.89		99.95	99.67	97.89
BWA	40:32	97.80	100.00	99.97	99.62	98.95	100.00	99.97	99.62	98.95	100.00	99.97	99.62
			94.26	83.53	75.30		93.82	79.03	70.93		93.82	79.16	71.17
GEM	3:15	96.02	98.25	98.24	98.09	98.15	98.27	98.18	98.01	99.35	99.42	99.41	99.24
			95.92	87.02	66.35		98.17	97.02	95.94		99.44	98.68	97.17
Masai	17:43	99.86	100.00	100.00	100.00	99.96	100.00	100.00	100.00	99.97	100.00	100.00	100.00
			99.87	99.54	97.87		99.85	99.29	98.59		99.85	98.34	98.70
Hobbes 2	7:51	99.82	99.98	99.97	99.97	99.97	99.98	99.97	99.97	99.99	100.00	99.99	99.98
			99.98	99.87	97.20		99.97	99.98	99.80		99.97	100.00	99.92
mrFAST	12:32	99.32	100.00	100.00	100.00	99.42	100.00	100.00	100.00	99.43	100.00	100.00	100.00
			100.00	99.96	87.51		100.00	100.00	53.69		100.00	100.00	54.09
RazerS 3 ^d^	41:33	99.92	100.00	100.00	100.00	99.99	100.00	100.00	100.00	99.99	100.00	100.00	100.00
			100.00	99.84	98.62		100.00	99.95	99.92		100.00	99.95	99.92
RazerS 3	54:59	100.00	100.00	100.00	100.00	100.00	100.00	100.00	100.00	100.00	100.00	100.00	100.00
			100.00	100.00	100.00		100.00	100.00	100.00		100.00	100.00	100.00
Yara	3:06	—	—	—	—	—	—	—	—	—	—	—	—
			—	—	—		—	—	—		—	—	—
BitMapper	2:57	99.99	100.00	100.00	100.00	100.00	100.00	100.00	100.00	100.00	100.00	100.00	100.00
			100.00	99.99	99.98		100.00	100.00	100.00		100.00	100.00	100.00

Bowtie2 ^a^, BWA ^b^, and GEM ^c^ represent the results in default sensitivity mode, while Bowtie2, BWA, and GEM represent the results in high sensitivity mode. The RazerS 3 ^d^ and RazerS 3 represent the results of RazerS 3 in default and full sensitivity mode, respectively. Note that in default mode, RazerS 3 is designed to find 99% of mapping locations, while Bowtie2, BWA, and GEM are designed to find the best mapping locations for each reads

Compared to the best-mappers, all-mappers usually achieve higher sensitivity. For mrFAST, it is interesting that its Rabema any-best and all-best scores were 53.69 % and 54.09 % at edit distance 5, which were much lower than other all-mappers. Masai and Hobbes 2 lost a few mapping locations due to their heuristic methods. BitMapper and RazerS 3 were the only two mappers identifying 100 % all of the best and any of the best mapping locations. Note that the all, all-best and any-best scores of RazerS 3 in full sensitivity mode were 100 %, since we used the output of RazerS 3 in full sensitivity mode as the baseline for Rabema benchmark. However, it was extremely slow. The Rabema all-score for BitMapper was nearly 100 %, which was the best except RazerS 3 in full sensitivity. We did not present the sensitivity of Yara in Table 1, since it could not generate CIGAR strings for suboptimal alignments, which led to incorrect output of Rabema benchmark.

#### Rabema benchmark results on real data

According to the results above, we found that the sensitivities of GEM and BWA on both high and default sensitivity modes were not high enough for the applications needing all or nearly all mapping locations. For Bowtie 2, although the sensitivity on high sensitivity mode has been improved, it spent much more time and memory than all-mappers. Thus, we would not present the results of them in the following.

To compare the sensitivity of all-mappers on real data sets, we also measured the Rabema scores using 1 million 100 bp reads of human, as shown in Table 2. And to evaluate the sensitivity for different genomes, the Rabema scores for caenorhabditis elegans genome and arabidopsis thaliana genome were presented in Tables 3 and 4, respectively. According to these results, we fonud that the sensitivity of Bitmapper was also best among all of the all-mappers except RazerS 3 in full sensitivity, which generated the baseline of Rabema benchmark. As the results in Table 1, the Rabema scores of Yara were not included in Tables 2, 3 and 4 due to the absence of CIGAR strings.

**Table 2:** Rabema benchmark results (*normalized found interval*) for 1 million 100 bp real reads of human

Mapper	Time	Benchmark category											
	[min:sec]	All[%]				All-best[%]				Any-best[%]			
Masai	42:50	99.94	100.00	100.00	100.00	99.99	100.00	100.00	100.00	99.99	100.00	100.00	100.00
			100.00	99.97	98.93		100.00	99.99	99.70		100.00	99.99	99.80
Hobbes 2	60:05	99.89	99.99	99.97	99.97	99.98	99.98	99.98	99.99	99.99	99.99	99.99	100.00
			99.96	99.91	98.43		99.99	99.99	99.91		100.00	100.00	99.99
mrFAST	98:06	99.79	100.00	100.00	100.00	99.91	100.00	100.00	100.00	99.92	100.00	100.00	100.00
			100.00	99.97	96.45		100.00	99.96	93.61		100.00	99.97	93.88
RazerS 3 ^a^	372:21	99.90	100.00	100.00	100.00	99.99	100.00	100.00	100.00	99.99	100.00	100.00	100.00
			100.00	99.80	98.45		100.00	99.90	99.47		100.00	99.91	99.70
RazerS 3	512:46	100.00	100.00	100.00	100.00	100.00	100.00	100.00	100.00	100.00	100.00	100.00	100.00
			100.00	100.00	100.00		100.00	100.00	100.00		100.00	100.00	100.00
Yara	29:25	—	—	—	—	—	—	—	—	—	—	—	—
			—	—	—		—	—	—		—	—	—
BitMapper	17:03	99.99	100.00	100.00	100.00	99.99	100.00	100.00	100.00	99.99	100.00	100.00	100.00
			100.00	99.99	99.98		100.00	100.00	99.98		100.00	100.00	99.99

RazerS 3 ^a^: the result of RazerS 3 in default sensitivity mode (i.e., finding 99% of mapping locations); RazerS 3: the result of RazerS 3 in full sensitivity mode (i.e., finding 100% of mapping locations)

**Table 3:** Rabema benchmark results (*normalized found interval*) for 1 million 100 bp real reads of caenorhabditis elegans

Mapper	Time	Benchmark category											
	[min:sec]	All[%]				All-best[%]				Any-best[%]			
Masai	3:02	99.93	100.00	100.00	100.00	99.99	100.00	100.00	100.00	99.99	100.00	100.00	100.00
			99.92	99.67	98.14		99.98	99.93	99.78		99.99	99.95	99.89
Hobbes 2	2:01	99.94	100.00	100.00	100.00	99.99	100.00	100.00	100.00	99.99	100.00	100.00	100.00
			99.99	99.86	98.18		99.99	99.96	99.72		99.99	99.99	99.97
mrFAST	3:40	98.89	100.00	100.00	100.00	99.95	100.00	100.00	100.00	99.96	100.00	100.00	100.00
			99.99	99.99	96.50		99.99	100.00	93.43		99.99	100.00	93.89
RazerS 3 ^a^	7:18	99.95	100.00	100.00	100.00	99.99	100.00	100.00	100.00	99.99	100.00	100.00	100.00
			99.99	99.79	98.64		99.99	99.89	99.61		99.99	99.95	99.84
RazerS 3	7:53	100.00	100.00	100.00	100.00	100.00	100.00	100.00	100.00	100.00	100.00	100.00	100.00
			100.00	100.00	100.00		100.00	100.00	100.00		100.00	100.00	100.00
Yara	1:25	—	—	—	—	—	—	—	—	—	—	—	—
			—	—	—		—	—	—		—	—	—
BitMapper	0:32	99.99	100.00	100.00	100.00	99.99	100.00	100.00	100.00	99.99	100.00	100.00	100.00
			100.00	99.99	99.98		100.00	99.99	99.98		100.00	99.99	99.98

RazerS 3 ^a^: the result of RazerS 3 in default sensitivity mode (i.e., finding 99% of mapping locations); RazerS 3: the result of RazerS 3 in full sensitivity mode (i.e., finding 100% of mapping locations)

**Table 4:** Rabema benchmark results (*normalized found interval*) for 1 million 101 bp real reads of arabidopsis thaliana

Mapper	Time	Benchmark category											
	[min:sec]	All[%]				All-best[%]				Any-best[%]			
Masai	3:05	99.96	100.00	100.00	100.00	99.99	100.00	100.00	100.00	99.99	100.00	100.00	100.00
			99.97	99.96	99.25		99.98	99.96	99.27		99.98	99.96	99.53
Hobbes 2	1:52	99.92	100.00	100.00	99.99	99.99	100.00	100.00	99.99	99.99	100.00	100.00	99.99
			99.97	99.88	98.72		99.98	99.99	99.64		99.99	100.00	99.98
mrFAST	2:30	99.88	100.00	100.00	100.00	99.98	100.00	100.00	100.00	99.99	100.00	100.00	100.00
			99.99	99.99	97.94		99.99	100.00	97.70		100.00	100.00	98.50
RazerS 3 ^a^	8:30	99.88	100.00	100.00	100.00	99.98	100.00	100.00	100.00	99.99	100.00	100.00	100.00
			99.99	99.70	98.12		99.98	99.70	98.63		99.99	99.84	99.27
RazerS 3	9:06	100.00	100.00	100.00	100.00	100.00	100.00	100.00	100.00	100.00	100.00	100.00	100.00
			100.00	100.00	100.00		100.00	100.00	100.00		100.00	100.00	100.00
Yara	1:25	—	—	—	—	—	—	—	—	—	—	—	—
			—	—	—		—	—	—		—	—	—
BitMapper	0:32	99.99	100.00	100.00	99.99	99.99	100.00	100.00	99.99	99.99	100.00	100.00	99.99
			99.98	100.00	99.99		99.99	100.00	99.99		99.99	100.00	99.99

RazerS 3 ^a^: the result of RazerS 3 in default sensitivity mode (i.e., finding 99% of mapping locations); RazerS 3: the result of RazerS 3 in full sensitivity mode (i.e., finding 100% of mapping locations)

### Performance comparison on large data sets

In order to compare the performance of BitMapper with other mappers on large data sets, we selected a single-end data set consisting of 10 million 100 bp reads from specimen HG00096 of the 1000 Genome project. And to compare the performance in different genomes, we used the first 10 million 100 bp reads of the data sets SRX026594 and the first 10 million 101 bp reads of SRR1604937, which were obtained from the DNA Data Bank of Japan (DDBJ) repository and National Center for Biotechnology Information (NCBI) repository, respectively. The first 10 million read pairs of these data sets were also used to measure the performance of paired-end alignment. Moreover, to demonstrate that Bitmapper also works well for longer reads, a real data set and two simulated data sets were used. The real data set consists of the first 10 million 151 bp reads of human in the HiSeq 2500 NA12878 demo data set in [23], while the two simulated data sets include 10 million 300 bp reads of caenorhabditis elegans and arabidopsis thaliana, respectively. All of these data sets with 100 bp, 151 bp and 300 bp reads were mapped to their reference genomes using edit distance threshold 5, 7 and 15, respectively.

Because the Rabema benchmark cannot be implemented in such large data sets, we used the percentage of mapped reads and the number of mapping sites to measure the sensitivity, instead of Rabema scores. For running time comparison, the results of different mappers with single and eight threads were presented in Tables 5, 6, 7 and 8. Note that since Masai and mrFAST do not support multi-threading, the results of them with eight threads were omitted. In addition, peak memory consumption was also compared.

**Table 5:** Results for mapping 10 million 100 bp and 151 bp single-end reads against human genome

	100 bp reads						151 bp reads					
Mapper	Time[min:sec]		Peak	Mapping	Mapped		Time [min:sec]		Peak	Mapping	Mapped	
	1 thr	8 thr	memory	sites[million]	reads[%]		1 thr	8 thr	memory	sites[million]	reads[%]	
Masai	361:35	—	20.1GB	1371.18	92.2736		602:06	—	21.3GB	939.89	93.8483	
Hobbes 2	587:04	135:10	14.0GB	1368.86	92.2767		694:53	151:52	14.5GB	936.42	93.8481	
mrFAST	921:46	—	4.9GB	1374.76	92.2572		795:59	—	6.5GB	939.48	93.7376	
RazerS 3 ^a^	—	—	>24GB	—	—		—	—	>24GB	—	—	
RazerS 3	—	—	>24GB	—	—		—	—	>24GB	—	—	
Yara	278:09	78:10	9.0GB	1367.42	92.2658		389:56	93:15	9.3GB	939.44	93.8480	
BitMapper	158:57	32:59	17.9GB	1375.68	92.2771		135:06	27:56	19.2GB	940.16	93.8487	

RazerS 3 ^a^: the result of RazerS 3 in default sensitivity mode (i.e., finding 99% of mapping locations); RazerS 3: the result of RazerS 3 in full sensitivity mode (i.e., finding 100% of mapping locations)

**Table 6:** Results for mapping 10 million 100 bp single-end reads against caenorhabditis elegans and arabidopsis thaliana

	Caenorhabditis elegans						Arabidopsis thaliana					
Mapper	Time [min:sec]		Peak	Mapping	Mapped		Time [min:sec]		Peak	Mapping	Mapped	
	1 thr	8 thr	memory	sites[million]	reads[%]		1 thr	8 thr	memory	sites[million]	reads[%]	
Masai	22:28	—	3.2GB	54.61	90.4140		21:06	—	3.3GB	57.83	98.2578	
Hobbes 2	16:51	4:42	0.9GB	55.40	90.4150		16:05	3:42	1.0GB	57.76	98.2616	
mrFAST	35:15	—	4.2GB	55.60	90.4119		23:12	—	4.3GB	57.94	98.2609	
RazerS 3 ^a^	69:24	59:39	12.0GB	55.24	90.4118		86:39	72:31	10.3GB	57.49	98.2551	
RazerS 3	75:27	61:40	12.6GB	55.61	90.4154		89:31	75:26	10.4GB	57.96	98.2622	
Yara	13:42	3:37	1.1GB	54.65	90.4150		15:13	4:01	1.2GB	57.87	98.2608	
BitMapper	5:08	1:25	4.5GB	55.63	90.4159		5:24	1:30	4.5GB	57.94	98.2631	

RazerS 3 ^a^: the result of RazerS 3 in default sensitivity mode (i.e., finding 99% of mapping locations); RazerS 3: the result of RazerS 3 in full sensitivity mode (i.e., finding 100% of mapping locations)

**Table 7:** Results for mapping 10 million 300bp single-end reads against caenorhabditis elegans and arabidopsis thaliana

	Caenorhabditis elegans						Arabidopsis thaliana					
Mapper	Time [min:sec]		Peak	Mapping	Mapped		Time [min:sec]		Peak	Mapping	Mapped	
	1 thr	8 thr	memory	sites[million]	reads[%]		1 thr	8 thr	memory	sites[million]	reads[%]	
Masai	48:54	—	11.5GB	17.44	99.9894		46:26	—	11.8GB	14.83	99.9884	
Hobbes 2	66:38	13:12	0.9GB	2.14	0.5327		64:25	12:50	1.0GB	0.01	0.0219	
mrFAST	80:56	—	9.9GB	16.71	96.1888		47:00	—	10.0GB	14.27	96.2356	
RazerS 3 ^a^	195:07	182:34	11.9GB	17.43	99.9894		172:21	155:48	12.0GB	14.82	99.9884	
RazerS 3	209:30	185:29	12.6GB	17.44	99.9894		185:05	160:02	12.0GB	14.83	99.9884	
Yara	34:20	7:43	2.1GB	17.33	99.9894		29:44	6:34	2.1GB	14.72	99.9884	
BitMapper	12:26	4:57	10.1GB	17.43	99.9894		12:10	4:55	10.2GB	14.83	99.9884	

RazerS 3 ^a^: the result of RazerS 3 in default sensitivity mode (i.e., finding 99% of mapping locations); RazerS 3: the result of RazerS 3 in full sensitivity mode (i.e., finding 100% of mapping locations)

**Table 8:** Results for mapping 10 million paired-end reads

	Human					Caenorhabditis elegans					Arabidopsis thaliana			
Mapper	Time [min:sec]		Peak	Mapped		Time [min:sec]		Peak	Mapped		Time [min:sec]		Peak	Mapped
	1 thr	8 thr	memory	pairs[%]		1 thr	8 thr	memory	pairs[%]		1 thr	8 thr	memory	pairs[%]
Masai	464:07	—	16.8GB	84.8984		31:07	—	11.3GB	65.8674		29:40	—	11.6GB	64.9149
Hobbes 2	439:05	105:29	14.6GB	87.3945		80:04	22:41	0.9GB	67.1739		23:59	6:21	1.0GB	68.1224
RazerS 3 ^a^	—	—	>24GB	—		61:15	47:11	16.4GB	67.1841		51:25	41:31	14.9GB	68.1250
RazerS 3	—	—	>24GB	—		66:28	50:13	17.4GB	67.1894		55:33	42:38	17.1GB	68.1473
Yara	489:58	117:40	13.2GB	87.1614		23:43	5:47	2.0GB	67.1058		28:09	6:52	2.2GB	66.8150
BitMapper	177:47	39:39	21.5GB	87.4233		11:16	3:15	8.0GB	67.1883		6:47	2:20	8.1GB	68.1500

RazerS 3 ^a^: the result of RazerS 3 in default sensitivity mode (i.e., finding 99% of mapping locations); RazerS 3: the result of RazerS 3 in full sensitivity mode (i.e., finding 100% of mapping locations)

#### Sensitivity and running time comparison

Table 5 shows the results of mapping 10 million 100 bp and 151 bp single-end reads to the whole human genome. Results of the best-mappers including GEM, Bowtie 2 and BWA were left out, because the sensitivity of them is usually substantially less than that of all-mappers and the running time is usually longer, as shown in Table 1 and Additional file 1: Table S3. The results in Table 5 show that BitMapper was the best in terms of sensitivity and running time on the human genome data sets. For 10 million 151 bp reads, it was nearly 3 times faster than the second fastest read mapper Yara, and achieved highest sensitivity with 940.16 million mapping locations identified and 93.8487 % reads mapped in the human genome. Compared to Masai, BitMapper was more than 4 times faster and found more mapping locations. For 10 million 100 bp reads, Bitmapper also presented the best performance among all read mappers. The results of RazerS 3 were not shown, since the memory requirement of RazerS 3 was larger than the memory capacity of our computer. Similarly, BitMapper was superior in mapping 10 million single-end reads against the genomes of caenorhabditis elegans and arabidopsis thaliana, as shown in Table 6. And for longer 300 bp reads, Bitmapper was still more efficient than others, as shown in Table 7.

Finally, Table 8 shows the experimental results for paired-end alignment, where three data sets consisting of 10 million read pairs from different genomes were used to evaluate the performance. Again, BitMapper was the best, 2.5 times faster than Hobbes 2, nearly 3 times faster than Masai and Yara in human genome. Note that the results of the human genome using RazerS 3 are not shown here, because the memory requirement of RazerS 3 was larger than the memory capacity of our computer. For caenorhabditis elegans and arabidopsis thaliana, BitMapper was also several times faster than the existing all-mappers. In addition, BitMapper still showed great performance in sensitivity comparison. We did not present the results of mrFAST, since it reported many extra locations. Thus, the running time was extremely long.

#### Memory usage comparison

If a reference genome is large, the memory usage of most mappers mainly depends on the size of the genome and the index for it. For instance, the human genome could be regarded as a long string including 3.15 billion symbols so that 3GB is required to store them. For hash table index, the locations for each *q*-gram should be saved and a location is represented by a 32-bit integer. Thus, BitMapper and Hobbes 2, which both index the reference genome using hash tables, require more than 14GB to load the index and genome. Similarly, Masai requires large memory space and uses about 20GB to map 10 million reads to human genome. Although the hash table index is also used in mrFAST, only 7GB is used since it splits the whole human genome and index into several segments and loads one of them at a time. Yara is another read mapper which requires small memory space, since it uses the BWT and FM-index. The memory usage of RazerS 3 mainly depends on the number of mapping locations. It needs more than 24GB to map 10 million 151 bp reads of human.

## Conclusion

BitMapper is designed to find all mapping locations for each read based on bit-vector computing. In experiments on both simulated and real data sets, it achieved nearly full sensitivity and superior speed, outperforming existing state-of-the-art all-mappers.

The verification of edit distance constitutes a significant portion of the whole running time. We propose a new vectorized Gene Myers’ bit-vector algorithm, which calculates the edit distance of a read to multiple locations in a given genome. To make full use of the algorithm, the traditional verification scheme is redesigned in BitMapper.

Recently, a new SIMD instruction set AVX2 has been applied to many CPUs. Thus, the performance of our vectorized Gene Myers’ bit-vector algorithm will be improved further by using AVX2 in the future. The vectorized bit-vector computing approach can also be used to accelerate filtration, which is a future research direction in BitMapper.

BitMapper is implemented in C under a GPL license and is able to download at http://home.ustc.edu.cn/%7Echhy.

## Additional file

Additional file 1 **Supplementary material.** This file consists of the configuration of each read mapper and the analysis of the two vectorized verification schemes. Besides, we present the pseudo code of our vectorized bit-vector algorithm and the performance comparison between it and other existing implementation of the Gene Myers’ algorithmin in the additional file.

## References

[1] Langmead B, Trapnell C, Pop M, Salzberg SL. Ultrafast and memory-efficient alignment of short dna sequences to the human genome. Genome Biol. 2009; 10(3):25. 10.1186/gb-2009-10-3-r25 PMC2690996 19261174

[2] Langmead B, Salzberg SL (2012). Fast gapped-read alignment with bowtie 2. Nat Methods.

[3] Li H, Durbin R (2009). Fast and accurate short read alignment with burrows–wheeler transform. Bioinformatics.

[4] Marco-Sola S, Sammeth M, Guigó R, Ribeca P (2012). The gem mapper: fast, accurate and versatile alignment by filtration. Nat Methods.

[5] Weese D, Holtgrewe M, Reinert K (2012). Razers 3: faster, fully sensitive read mapping. Bioinformatics.

[6] Kim J, Li C, Xie X. Improving read mapping using additional prefix grams. BMC Bioinformatics. 2014; 15(1):42. 10.1186/1471-2105-15-42 PMC3927682 24499321

[7] Hach F, Hormozdiari F, Alkan C, Hormozdiari F, Birol I, Eichler EE (2010). mrsfast: a cache-oblivious algorithm for short-read mapping. Nat Methods.

[8] Xin H, Lee D, Hormozdiari F, Yedkar S, Mutlu O, Alkan C. Accelerating read mapping with fasthash. BMC Genomics. 2013; 14(Suppl 1):13. 10.1186/1471-2164-14-S1-S13 PMC3549798 23369189

[9] Ahmadi A, Behm A, Honnalli N, Li C, Weng L, Xie X (2012). Hobbes: optimized gram-based methods for efficient read alignment. Nucleic Acids Res.

[10] Siragusa E, Weese D, Reinert K (2013). Fast and accurate read mapping with approximate seeds and multiple backtracking. Nucleic Acids Res.

[11] Myers G (1999). A fast bit-vector algorithm for approximate string matching based on dynamic programming. J ACM (JACM).

[12] Hyyrö H (2003). A bit-vector algorithm for computing levenshtein and damerau edit distances. Nord J Comput.

[13] Siragusa WD E, Reinert K. Yara: well-defined alignment of high-throughput sequencing reads. http://www.seqan.de/projects/yara/.

[14] Rasmussen KR, Stoye J, Myers EW (2006). Efficient q-gram filters for finding all *ε*-matches over a given length. J Comput Biol.

[15] Weese D, Emde AK, Rausch T, Döring A, Reinert K (2009). Razers-fast read mapping with sensitivity control. Genome Res.

[16] Sellers PH (1980). The theory and computation of evolutionary distances: pattern recognition. J Algorithms.

[17] Ukkonen E (1985). Finding approximate patterns in strings. J Algorithms.

[18] Holtgrewe M. Mason–a read simulator for second generation sequencing data. Technical Report FU Berlin. 2010.

[19] 1000 Genomes: a Deep Catalog of Human Genetic Variation. http://www.1000genomes.org/data.

[20] DNA Data Bank of Japan. ftp://ftp.ddbj.nig.ac.jp.

[21] National Center for Biotechnology Information. http://www.ncbi.nlm.nih.gov/.

[22] Holtgrewe M, Emde AK, Weese D, Reinert K. A novel and well-defined benchmarking method for second generation read mapping. BMC Bioinformatics. 2011; 12(1):210. 10.1186/1471-2105-12-210 PMC3128034 21615913

[23] BaseSpace Sequencing Data Sets. http://www.illumina.com/informatics/research/sequencing-data-analysis-management/sequencing-data-library.html.

