# Supplementary material for: BitMapper: an efficient all-mapper based on bit-vector computing
Source: BMC Bioinformatics. 2015 Jun 11;16:192. doi: 10.1186/s12859-015-0626-9 (PMC4462005; doi:10.1186/s12859-015-0626-9)
Supplement: Supplementary file 1 — Supplementary material. This file consists of the configuration of each read mapper and the analysis of the two vectorized verification schemes. Besides, we present the pseudo code of our vectorized bit-vector algorithm and the performance comparison between it and other existing implementation of the Gene Myers’ algorithmin in the additional file. [file 12859_2015_626_MOESM1_ESM.pdf]

# Supplementary Material for

"BitMapper: An efficient all-mapper based on bit-vector computing"

June 8, 2015

## S1 Configuration of each read mapper

In our experiments, we configured each read mapper as follows.

**Masai**.Version 0.7.2 was used. We passed the parameter -mm all to Masai to find all mapping locations. For paired-end alignment, the parameters -ll and -le were used. The thresholds of edit distance were set to 5, 7 and 15 for different data sets. To generate the results, Masai needs to produce raw outputs and convert them into SAM format outputs separately. The running time of Masai consisted of these two parts.

**Hobbes 2**.Version 2.0 was used. The -a option was used to output all mapping locations. We also used −−indel and -v options to set the threshold of edit distance. The −−min and −−max options were used in paired-end model. Because the outputs of Hobbes 2 could not directly be used by Rabema benchmark, we manually processed it to meet the requirements of Rabema benchmark.

**mrFAST**.Version 2.6.0.2 was used. Like Hobbes 2, the outputs of mrFAST can not used by Rabema benchmark. Thus, we manually processed the outputs of it.

**RazerS 3**. Version 3.2 was used. The -m 1,000,000 option was used to generate all mapping locations for each read. In paired-end mode, the -ll and -le options were used. Besides, we used the default parameters of RazerS3.

**BitMapper**. Version 1.0.0.6 was used. To set the threshold of edit distance, we passed the parameter -e to BitMapper. For paired-end alignment, the −−min and −−max options were used.

**Yara**. Version 0.9.0 was used. The threshold of edit distance was set to 5%. We used -os and -a to output all alignments.

**Bowtie 2**. Version 2.2.3 was used. The -a option was used to configure Bowtie2 as an all-mapper. To set the threshold of edit distance to 5, the options −−mp , −−np, −−rdg, −−rfg , and −−score-min parameters were configured to [1,1], 1, [0,1], [0,1], and [L,0, 0.05], respectively. We also used the parameters −−end-to-end and −−ignore-quals to focus on the edit distance metric.

**BWA**. Version 0.7.12 was used. We used the -N option to configure BWA as an all-mapper. The parameters -n, -o, and -e were set to 5, 5, and -1, respectively. We also used BWA in default mode to find the best mapping locations.

**GEM**. Version 1.376 was used. The -q ignore option was used to ignore the quality and focus on the edit distance metric. We set the threshold of edit distance by configuring -m and -e parameters to 5. To configure GEM as an all mapper, we set -d parameter to all and -s parameter to 1,000,000. Like Masai, we need to convert the raw outputs into SAM format outputs after mapping. The conversion time was also included in the running time.

Table S1: The proportion of the candidate locations which were verified with a read group by group in Bitmapper. Each group includes $n$ locations.

| Genome | n=1 | n=2 | n=3 | n=4 | n=5 | n=6 | n=7 | n=8 |
|---|---|---|---|---|---|---|---|---|
| Caenorhabditis elegans | 3.82% | 0.77% | 0.57% | 0.64% | 0.69% | 0.76% | 0.82% | 91.95% |
| Arabidopsis thaliana | 1.32% | 0.87% | 0.74% | 0.82% | 0.99% | 1.12% | 1.26% | 92.88% |
| Human | 0.02% | 0.01% | 0.01% | 0.01% | 0.01% | 0.01% | 0.01% | 99.89% |

Table S2: The proportion of 11-grams which are shared with $m$ read in different data sets.

| Reads number | $m$=1 | $m$=2 | $m$=3 | $m$=4 | $m\geq5$ |
|---|---|---|---|---|---|
| 1 million | 92.59% | 5.97% | 0.81% | 0.26% | 0.37% |
| 2 million | 88.21% | 9.33% | 1.45% | 0.41% | 0.61% |
| 3 million | 84.42% | 12.00% | 2.18% | 0.59% | 0.81% |
| 4 million | 81.04% | 14.17% | 2.97% | 0.82% | 1.00% |
| 5 million | 77.98% | 15.95% | 3.77% | 1.09% | 1.21% |

# S2 Comparison of two vectorized verification schemes

To make full use of our vectorized Gene Myers' bit-vector algorithm, two vectorized verification schemes are proposed named as scheme A and scheme B. The former utilizes the repeatability of reads, while the latter utilizes the repeatability of the reference genome. Therefore, if BitMapper uses scheme A, the speedup of it mainly depends on the number of the reads sharing same locations. If BitMapper uses scheme B, the speedup mainly depends on the number of the candidate locations for each read.

Table S1 shows the proportion of the candidate locations which were processed group by group. Two data sets consisting of 1 million 100bp reads were mapped to the whole genome of human (NCBI HG19) and caenorhabditis elegans (WormBase WS201), respectively. Another data set consisting of 1 million 101bp reads of arabidopsis thaliana (assembly TAIR10) was also used. The size of human genome is nearly 30 times larger than the others. Even for the smallest genome of caenorhabditis elegans, 91.95% candidate locations were processed eight by eight. For human genome, the proportion was nearly 100%. It means that using scheme B is able to make full use of our vectorized Gene Myers' bit-vector algorithm.

In order to use scheme A, first build an extra index for reads. Given a reads data set $R$ and an edit distance threshold $k$, partition each read in $R$ into $k$+2 non-overlapping 11-grams, according to the variant of pigeonhole filter used in BitMapper. Then we create a hash table index $RI$. The hash keys for each entry in $RI$ are a 11-gram $g$ and a offset $c$, and the hash value is a list of reads which consist of $g$ at $c$. Thus, each location in the inverted list of a 11-gram should be verified with the reads which correspond to this 11-gram in $RI$.

Observation from Table S2, the repeatability of the reads was much lower than that of the reference genomes. In the experiment, each read and its reverse-complement counterpart were partitioned into seven non-overlapping 11-grams. Even for the largest data set with 5 million 100bp reads, only 1.21% 11-grams were shared with more than 4 reads. However, if scheme A is used in BitMapper, our vectorized Gene Myers' bit-vector algorithm needs eight reads to process simultaneously. Thus, the performance of scheme A was not as well as scheme B.

## S3 Performance comparison of different bit-vector algorithms

We ran an implementation of the Gene Myers' algorithm provided in SeqAn library [1] to compare the performance of it with our vectorized Gene Myers' bit-vector algorithm. SeqAn is an open source C++ library designed for the biological analysis algorithms. It is efficient and has been widely used. In this experiment, we selected a 100bp read from specimen HG00096 as a text and regarded 1 thousand subsequences of human genome starting at the candidate locations of this read as patterns. The threshold $k$ of edit distance was set to 4.

Figure S1 shows the results. Besides the result of SeqAn's implementation, we only presented the results of 1-vectorized algorithm (the vectorized algorithm which processes 1 pattern simultaneously), 2-vectorized algorithm (the vectorized algorithm which processes 2 patterns simultaneously), 4-vectorized algorithm (the vectorized algorithm which processes 4 patterns simultaneously), and 8-vectorized algorithm (the vectorized algorithm which processes 8 patterns simultaneously) here. In fact, only these algorithms are used in Bitmapper. According to the results, we found that the running time of the SeqAn's implementation was similar to that of the 1-vectorized algorithm. For other vectorized algorithms, the the running time was greatly reduced.

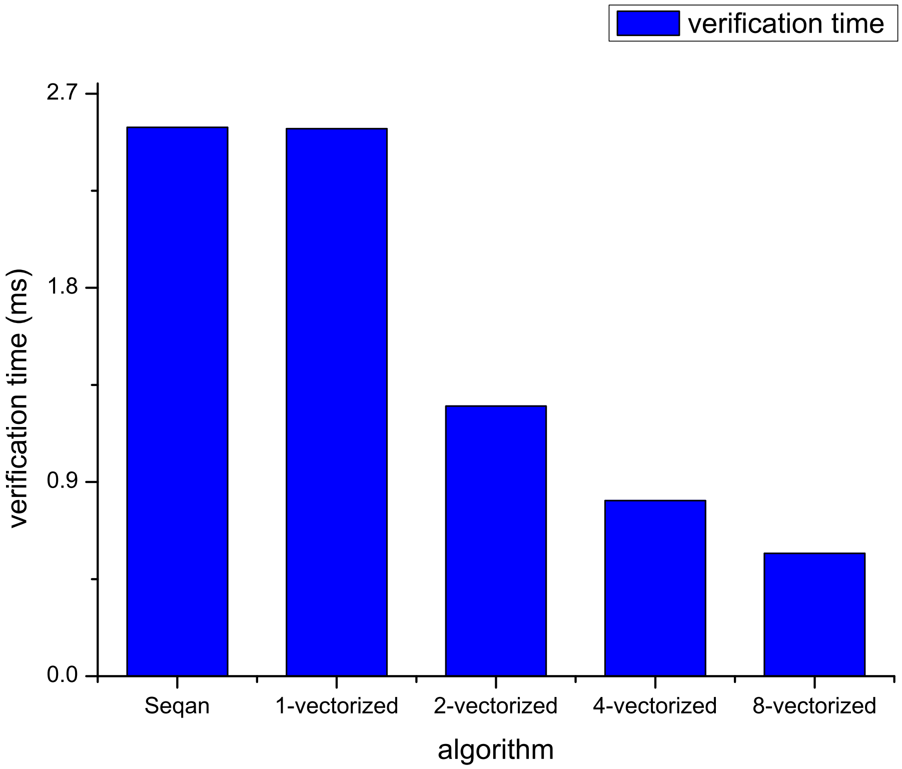


Figure S1: Performance comparison between the vectorized Gene Myers' bit-vector algorithm and the SeqAn's implementation.

# S4 Rabema benchmark results (*found interval*)

In order to measure the Rabema scores, Rabema benchmark defines two metrics: *normalized found interval* and *found interval* [2]. The definitions of them are as flowing.

1. **Normalized found interval:** Each read gives at most one point no matter how many mapping locations it has. If a read identify *n* mapping locations (i.e. intervals), each one gives 1/*n* point.

2. **Found interval:** The point of each read is given according to the number of its mapping locations. If a read identify *n* mapping locations (i.e. intervals), each found location gives one point. Thus, this read would give at most *n* point.

Table S3 shows the Rabema scores (*found interval*) for 100k simulated reads of human. The Rabema all-scores (*found interval*) for BWA (high sensitivity mode) and GEM (high sensitivity mode) were 86.67% and 61.46% respectively, while the Rabema all-scores (*found interval*) for most of all-mappers were more than 99%. Thus, the sensitivity of BWA and GEM is not enough for the applications requiring full or nearly full sensitivity. Although Bowtie 2 in high sensitivity identified more than 98% mapping locations, it required much more time and memory than all-mappers. Because of this, we omitted the results of it in Table S4, Table S5 and Table S6.

Table S3: Rabema scores (*found interval*) for 100k simulated reads of human

| Mapper | Match | Benchmark category | | | | | | | | | | | |
|---|---|---|---|---|---|---|---|---|---|---|---|---|---|
| | [million] | All[%] | | | | All-best[%] | | | | Any-best[%] | | | |
| Bowtie2* | 0.10 | 0.74 | 25.31<br>0.16 | 5.26<br>0.05 | 0.86<br>0.02 | 30.78 | 25.31<br>35.41 | 36.47<br>65.02 | 40.16<br>58.12 | 99.26 | 100.00<br>96.63 | 99.49<br>96.27 | 97.51<br>95.38 |
| BWA* | 0.61 | 4.61 | 100.00<br>1.30 | 43.09<br>0.17 | 8.38<br>0.06 | 92.95 | 100.00<br>96.49 | 87.84<br>60.10 | 70.49<br>47.06 | 98.89 | 100.00<br>93.70 | 99.86<br>78.61 | 99.50<br>71.17 |
| GEM* | 0.32 | 2.37 | 97.27<br>0.58 | 12.88<br>0.28 | 1.55<br>0.20 | 89.79 | 97.27<br>72.43 | 88.10<br>78.33 | 58.18<br>71.39 | 99.36 | 99.42<br>99.42 | 99.42<br>98.74 | 99.24<br>97.33 |
| Bowtie 2 | 13.17 | 98.73 | 100.00<br>100.00 | 100.00<br>99.74 | 100.00<br>96.79 | 99.99 | 100.00<br>99.98 | 100.00<br>99.74 | 100.00<br>98.57 | 99.97 | 100.00<br>99.95 | 100.00<br>99.67 | 100.00<br>97.89 |
| BWA | 11.99 | 86.67 | 100.00<br>96.94 | 100.00<br>88.03 | 99.67<br>74.70 | 99.37 | 100.00<br>97.43 | 99.99<br>78.62 | 99.73<br>47.76 | 98.95 | 100.00<br>93.82 | 99.97<br>79.16 | 99.62<br>71.17 |
| GEM | 8.23 | 61.46 | 97.27<br>66.38 | 92.69<br>57.74 | 86.26<br>49.41 | 88.75 | 97.27<br>75.73 | 83.79<br>76.92 | 58.11<br>85.89 | 99.35 | 99.42<br>99.44 | 99.41<br>98.68 | 99.24<br>97.17 |
| Masai | 13.24 | 99.30 | 100.00<br>99.99 | 100.00<br>99.91 | 100.00<br>98.19 | 99.97 | 100.00<br>99.94 | 100.00<br>97.85 | 100.00<br>98.91 | 99.97 | 100.00<br>99.85 | 100.00<br>98.34 | 100.00<br>98.70 |
| Hobbes 2 | 13.19 | 98.74 | 99.93<br>99.96 | 99.94<br>99.72 | 99.95<br>96.88 | 99.93 | 99.93<br>99.94 | 99.94<br>99.56 | 99.94<br>99.70 | 99.99 | 100.00<br>99.97 | 99.99<br>100.00 | 99.98<br>99.92 |
| mrFAST | 13.33 | 99.24 | 100.00<br>100.00 | 100.00<br>99.98 | 100.00<br>97.99 | 99.81 | 100.00<br>100.00 | 100.00<br>100.00 | 100.00<br>69.76 | 99.43 | 100.00<br>100.00 | 100.00<br>100.00 | 100.00<br>54.09 |
| RazerS 3* | 13.24 | 99.25 | 100.00<br>99.99 | 100.00<br>99.70 | 100.00<br>98.21 | 99.99 | 100.00<br>100.00 | 100.00<br>99.96 | 100.00<br>99.95 | 99.99 | 100.00<br>100.00 | 100.00<br>99.95 | 100.00<br>99.92 |
| RazerS 3 | 13.34 | 100.00 | 100.00<br>100.00 | 100.00<br>100.00 | 100.00<br>100.00 | 100.00 | 100.00<br>100.00 | 100.00<br>100.00 | 100.00<br>100.00 | 100.00 | 100.00<br>100.00 | 100.00<br>100.00 | 100.00<br>100.00 |
| Yara | 13.17 | — | —<br>— | —<br>— | —<br>— | — | —<br>— | —<br>— | —<br>— | — | —<br>— | —<br>— | —<br>— |
| BitMapper | 13.34 | 99.99 | 100.00<br>100.00 | 100.00<br>99.99 | 100.00<br>99.98 | 100.00 | 100.00<br>100.00 | 100.00<br>100.00 | 100.00<br>100.00 | 100.00 | 100.00<br>100.00 | 100.00<br>100.00 | 100.00<br>100.00 |

Bowtie2*, BWA*, and GEM* represent the results in default sensitivity mode, while Bowtie2, BWA, and GEM represent the results in high sensitivity mode.

Table S4: Rabema scores (*found interval*) for 1 million 100bp real reads of human

| Mapper | Match | Benchmark category | | | | | | | | | | | |
|---|---|---|---|---|---|---|---|---|---|---|---|---|---|
| | [million] | All[%] | | | | All-best[%] | | | | Any-best[%] | | | |
| Masai | 136.62 | 99.69 | 100.00<br>100.00 | 100.00<br>99.98 | 100.00<br>99.13 | 99.99 | 100.00<br>100.00 | 100.00<br>99.99 | 100.00<br>99.67 | 99.99 | 100.00<br>100.00 | 100.00<br>99.99 | 100.00<br>99.80 |
| Hobbes 2 | 136.35 | 99.41 | 99.93<br>99.96 | 99.94<br>99.88 | 99.96<br>98.44 | 99.93 | 99.93<br>99.98 | 99.95<br>99.97 | 99.97<br>99.71 | 99.99 | 99.99<br>100.00 | 99.99<br>100.00 | 100.00<br>99.99 |
| mrFAST | 136.97 | 99.25 | 100.00<br>100.00 | 100.00<br>99.98 | 100.00<br>97.84 | 99.96 | 100.00<br>99.99 | 100.00<br>99.84 | 100.00<br>94.09 | 99.92 | 100.00<br>100.00 | 100.00<br>99.97 | 100.00<br>93.88 |
| RazerS 3* | 135.96 | 99.22 | 100.00<br>99.97 | 100.00<br>99.66 | 100.00<br>98.01 | 99.99 | 100.00<br>100.00 | 100.00<br>99.89 | 100.00<br>99.13 | 99.99 | 100.00<br>100.00 | 100.00<br>99.91 | 100.00<br>99.70 |
| RazerS 3 | 137.03 | 100.00 | 100.00<br>100.00 | 100.00<br>100.00 | 100.00<br>100.00 | 100.00 | 100.00<br>100.00 | 100.00<br>100.00 | 100.00<br>100.00 | 100.00 | 100.00<br>100.00 | 100.00<br>100.00 | 100.00<br>100.00 |
| Yara | 136.22 | — | —<br>— | —<br>— | —<br>— | — | —<br>— | —<br>— | —<br>— | — | —<br>— | —<br>— | —<br>— |
| BitMapper | 137.02 | 99.99 | 100.00<br>100.00 | 100.00<br>99.99 | 100.00<br>99.99 | 99.99 | 100.00<br>100.00 | 100.00<br>99.90 | 100.00<br>99.83 | 99.99 | 100.00<br>100.00 | 100.00<br>100.00 | 100.00<br>99.99 |

RazerS 3*: the result of RazerS 3 in default sensitivity mode (i.e., finding 99% of mapping locations); RazerS 3: the result of RazerS 3 in full sensitivity mode (i.e., finding 100% of mapping locations).

Table S5: Rabema scores (*found interval*) for 1 million 100bp real reads of caenorhabditis elegans

| Mapper | Match | Benchmark category | | | | | | | | | | | |
|---|---|---|---|---|---|---|---|---|---|---|---|---|---|
| | [million] | All[%] | | | | All-best[%] | | | | Any-best[%] | | | |
| Masai | 5.63 | 98.10 | 100.00<br>99.13 | 100.00<br>97.92 | 100.00<br>94.69 | 99.99 | 100.00<br>99.97 | 100.00<br>99.85 | 100.00<br>99.59 | 99.99 | 100.00<br>99.99 | 100.00<br>99.95 | 100.00<br>99.89 |
| Hobbes 2 | 5.71 | 99.31 | 100.00<br>100.00 | 100.00<br>99.82 | 100.00<br>97.52 | 99.99 | 100.00<br>99.98 | 100.00<br>99.96 | 100.00<br>99.24 | 99.99 | 100.00<br>99.99 | 100.00<br>99.99 | 100.00<br>99.97 |
| mrFAST | 5.73 | 99.38 | 100.00<br>100.00 | 100.00<br>99.98 | 100.00<br>97.66 | 99.94 | 100.00<br>99.99 | 100.00<br>100.00 | 100.00<br>95.11 | 99.96 | 100.00<br>99.99 | 100.00<br>100.00 | 100.00<br>93.89 |
| RazerS 3* | 5.70 | 99.33 | 100.00<br>99.99 | 100.00<br>99.55 | 100.00<br>97.82 | 99.97 | 100.00<br>99.99 | 100.00<br>99.25 | 100.00<br>98.66 | 99.99 | 100.00<br>99.99 | 100.00<br>99.95 | 100.00<br>99.84 |
| RazerS 3 | 5.74 | 100.00 | 100.00<br>100.00 | 100.00<br>100.00 | 100.00<br>100.00 | 100.00 | 100.00<br>100.00 | 100.00<br>100.00 | 100.00<br>100.00 | 100.00 | 100.00<br>100.00 | 100.00<br>100.00 | 100.00<br>100.00 |
| Yara | 5.59 | — | —<br>— | —<br>— | —<br>— | — | —<br>— | —<br>— | —<br>— | — | —<br>— | —<br>— | —<br>— |
| BitMapper | 5.74 | 99.99 | 100.00<br>100.00 | 100.00<br>99.99 | 100.00<br>99.98 | 99.99 | 100.00<br>99.99 | 100.00<br>99.99 | 100.00<br>99.99 | 99.99 | 100.00<br>100.00 | 100.00<br>99.99 | 100.00<br>99.98 |

RazerS 3*: the result of RazerS 3 in default sensitivity mode (i.e., finding 99% of mapping locations); RazerS 3: the result of RazerS 3 in full sensitivity mode (i.e., finding 100% of mapping locations).

Table S6: Rabema scores (*found interval*) for 1 million 101bp real reads of arabidopsis thaliana

| Mapper | Match | Benchmark category | | | | | | | | | | | |
|---|---|---|---|---|---|---|---|---|---|---|---|---|---|
| | [million] | All[%] | | | | All-best[%] | | | | Any-best[%] | | | |
| Masai | 5.83 | 99.79 | 100.00 | 100.00 | 100.00 | 99.98 | 100.00 | 100.00 | 100.00 | 99.99 | 100.00 | 100.00 | 100.00 |
| | | | 100.00 | 99.98 | 99.40 | | 99.99 | 99.99 | 99.45 | | 99.98 | 99.96 | 99.53 |
| Hobbes 2 | 5.82 | 99.58 | 100.00 | 100.00 | 100.00 | 99.98 | 100.00 | 100.00 | 100.00 | 99.99 | 100.00 | 100.00 | 99.99 |
| | | | 100.00 | 99.97 | 98.82 | | 99.99 | 99.98 | 99.34 | | 99.99 | 100.00 | 99.98 |
| mrFAST | 5.84 | 99.31 | 100.00 | 100.00 | 100.00 | 99.94 | 100.00 | 100.00 | 100.00 | 99.99 | 100.00 | 100.00 | 100.00 |
| | | | 100.00 | 100.00 | 97.99 | | 100.00 | 100.00 | 97.94 | | 100.00 | 100.00 | 98.50 |
| RazerS 3* | 5.79 | 99.17 | 100.00 | 100.00 | 100.00 | 99.93 | 100.00 | 100.00 | 100.00 | 99.99 | 100.00 | 100.00 | 100.00 |
| | | | 99.99 | 99.69 | 97.81 | | 99.99 | 99.58 | 98.39 | | 99.99 | 99.84 | 99.27 |
| RazerS 3 | 5.84 | 100.00 | 100.00 | 100.00 | 100.00 | 100.00 | 100.00 | 100.00 | 100.00 | 100.00 | 100.00 | 100.00 | 100.00 |
| | | | 100.00 | 100.00 | 100.00 | | 100.00 | 100.00 | 100.00 | | 100.00 | 100.00 | 100.00 |
| Yara | 5.71 | — | — | — | — | — | — | — | — | — | — | — | — |
| | | | — | — | — | | — | — | — | | — | — | — |
| BitMapper | 5.84 | 99.99 | 100.00 | 100.00 | 100.00 | 99.99 | 100.00 | 100.00 | 100.00 | 99.99 | 100.00 | 100.00 | 99.99 |
| | | | 100.00 | 100.00 | 100.00 | | 100.00 | 100.00 | 100.00 | | 99.99 | 100.00 | 99.99 |

RazerS 3*: the result of RazerS 3 in default sensitivity mode (i.e., finding 99% of mapping locations); RazerS 3: the result of RazerS 3 in full sensitivity mode (i.e., finding 100% of mapping locations).

# S5 Vectorized Gene Myers' bit-vector algorithm

**Algorithm 1** Vectorized Algorithm (*n*, *k*, *p*, *t*, *lt*, *lp*, *Dm*, *RDm*, *Em*, *RPm*, *Mask*, *VPm*)

**Input:** *n* is the number of patterns; *k* is the edit distance threshold; *p*[0...*n*-1] is an array of patterns; *t* is a text; *lt* is the length of text *t*; *lp* is the length of patterns of *p*; *Dm*, *RDm*, *Em*, *RPm*, *VPm* and *Mask*[0...*n*-1] are the bit masks for corresponding operations

**Output:** The matched optimal end positions *Sites*[0..*n*-1] and edit distance *Dis*[0..*n*-1] for the text in each patterns.

```
for all x ∈ {A, T, G, C, N} do                    //initialize the Peq array for n patterns
   Peq[x] ← 0
   for j ← 0 to n − 1 do
      for i ← 2k to 0 do                          //process the first 2k+1 symbols in each pattern
         Peq[x] ← Peq[x] ≪ 1
         if p[j][i] = x then
            Peq[x] ← Peq[x] + 1
         end if
      end for
      Peq[x] ← Peq[x] ≪ 1
   end for
end for
VP ← 0
VN ← 0
w ← 2k + 1
Pre_end ← lp − lt + k

for i ← 0 to lt − 1 do
   X ← Peq[t[i]] | VN                             //compute HP, HN and D0 using VP and VN
   M ← X & VP
   M ← ((M & Dm) + (VP & Dm))
   D0 ← (M ⊕ VP) | X
   HN ← VP & D0
   HP ← VN |∼ (VP | D0)
   X ← (D0 ≫ 1) & RDm
   VN ← X & HP                                    //compute VP and VN by HP, HN and D0
   VP ← HN &∼ (X | HP)
```

$E \leftarrow E + Em - (Em \,\&\, D0)$ //branch-cut strategy
$tmpE \leftarrow E$
**for** $j \leftarrow 0$ **to** $n-1$ **do**
  **if** $(tmpE \,\&\, 0^{64-2k-1}1^{2k+1}) \leq Pre\_end$ **then**
    **break**
  **end if**
  $tmpE \leftarrow tmpE \gg (2k+2)$
**end for**
**if** $j = n$ **then**
  **report not found**
**end if**
**for all** $x \in \{A, T, G, C\}$ **do** //shift the *Peq* array for *n* patterns
  $Peq[x] \leftarrow (Peq[x] \gg 1) \,\&\, RPm$
**end for**
**if** $(i + 2k - 1) < lt$ **then**
  **for** $j \leftarrow 0$ **to** $n-1$ **do**
    $Peq[p[j]] \leftarrow Peq[p[j]] \mid Mask[j]$
  **end for**
**end if**
**end for**

$loc \leftarrow 2lp - lt - 1$ //find the matched optimal location and the edit distance for each pattern
$tmpE \leftarrow E$
**for** $j \leftarrow 0$ **to** $n-1$ **do**
  $Dis[j] \leftarrow (tmpE \,\&\, 0^{64-2k-1}1^{2k+1})$
  **if** $Dis[j] < k$ **then**
    $Sites[j] \leftarrow loc$
  **end if**
  $tmpE \leftarrow tmpE \gg (2k+2)$
**end for**
$j \leftarrow 0$
**while** $j < lp - lt$ **do**
  $tmpVP \leftarrow VP \gg j$
  $tmpVP \leftarrow tmpVP \,\&\, VPm$
  $E \leftarrow E + tmpVP$
  $tmpVN \leftarrow VN \gg j$
  $tmpVN \leftarrow tmpVN \,\&\, VPm$
  $E \leftarrow E + tmpVN$
  $j \leftarrow j + 1$
  $tmpE \leftarrow E$
  **for** $i \leftarrow 0$ **to** $n-1$ **do**
    $Dis[i] \leftarrow (tmpE \,\&\, 0^{64-2k-1}1^{2k+1})$
    **if** $Dis[i] < k$ **then**
      $Sites[i] \leftarrow loc$
    **end if**
    $tmpE \leftarrow tmpE \gg (2k+2)$
  **end for**
**end while**

# References

[1] Döring, A., Weese, D., Rausch, T., Reinert, K.: Seqan an efficient, generic c++ library for sequence analysis. BMC bioinformatics **9**(1), 11 (2008)

[2] Holtgrewe, M., Emde, A.-K., Weese, D., Reinert, K.: A novel and well-defined benchmarking method for second generation read mapping. BMC bioinformatics **12**(1), 210 (2011)
